# Light Quality Modulates Plant Cold Response and Freezing Tolerance

**DOI:** 10.3389/fpls.2022.887103

**Published:** 2022-06-09

**Authors:** Michaela Kameniarová, Martin Černý, Jan Novák, Vladěna Ondrisková, Lenka Hrušková, Miroslav Berka, Radomira Vankova, Bretislav Brzobohatý

**Affiliations:** ^1^Department of Molecular Biology and Radiobiology, Faculty of AgriSciences, Mendel University in Brno, Brno, Czechia; ^2^Laboratory of Hormonal Regulations in Plants, Institute of Experimental Botany, The Czech Academy of Sciences, Prague, Czechia; ^3^Central European Institute of Technology, Faculty of AgriSciences, Mendel University in Brno, Brno, Czechia; ^4^Institute of Biophysics of the Czech Academy of Sciences, Brno, Czechia

**Keywords:** *Arabidopsis thaliana* (*Arabidopsis*), accession, cold, freezing tolerance, light intensity, light quality, photosynthesis, proteome

## Abstract

The cold acclimation process is regulated by many factors like ambient temperature, day length, light intensity, or hormonal status. Experiments with plants grown under different light quality conditions indicate that the plant response to cold is also a light-quality-dependent process. Here, the role of light quality in the cold response was studied in 1-month-old *Arabidopsis thaliana* (Col-0) plants exposed for 1 week to 4°C at short-day conditions under white (100 and 20 μmol m^−2^s^−1^), blue, or red (20 μmol m^−2^s^−1^) light conditions. An upregulated expression of *CBF1*, inhibition of photosynthesis, and an increase in membrane damage showed that blue light enhanced the effect of low temperature. Interestingly, cold-treated plants under blue and red light showed only limited freezing tolerance compared to white light cold-treated plants. Next, the specificity of the light quality signal in cold response was evaluated in *Arabidopsis* accessions originating from different and contrasting latitudes. In all but one *Arabidopsis* accession, blue light increased the effect of cold on photosynthetic parameters and electrolyte leakage. This effect was not found for Ws-0, which lacks functional CRY2 protein, indicating its role in the cold response. Proteomics data confirmed significant differences between red and blue light-treated plants at low temperatures and showed that the cold response is highly accession-specific. In general, blue light increased mainly the cold-stress-related proteins and red light-induced higher expression of chloroplast-related proteins, which correlated with higher photosynthetic parameters in red light cold-treated plants. Altogether, our data suggest that light modulates two distinct mechanisms during the cold treatment - red light-driven cell function maintaining program and blue light-activated specific cold response. The importance of mutual complementarity of these mechanisms was demonstrated by significantly higher freezing tolerance of cold-treated plants under white light.

## Introduction

Low temperature is one of the main factors limiting plant growth and overall crop production. However, plants that originate from various geographical areas have developed different levels of cold tolerance (Chen et al., 2014). Temperate climate plants can optimize their metabolism and growth in response to low temperatures in a process known as cold acclimation (Thomashow, 1999).

The primary targets of cold stress damage are cell membranes (reviewed in Mukhopadhyay and Roychoudhury, 2018). Low temperature induces changes in the composition of membranes and has a direct impact on their biophysical properties (Örvar et al., 2000; Palma et al., 2008; reviewed in Raju et al., 2018, and Yu et al., 2021). Tissue damages induced by freezing are characterized by loss of membrane integrity and higher permeability for molecules that can be followed and quantified by electrolyte leakage assay (Thalhammer et al., 2020). The first and most severely affected organelles in plants by cold stress are chloroplasts (reviewed in Liu et al., 2018a). Low temperature has an inhibitory effect on photosynthesis and promotes reactive oxygen species (ROS) generation (Liu et al., 2012; Zhang et al., 2020). ROS are toxic byproducts of inevitable processes like photosynthesis or respiration, and plants modulate their levels during cold stress (Luo et al., 2020; Lv et al., 2020). However, ROS also play an important role in mediating response to cold stress. Plant pre-treatment with scavenger of hydrogen peroxide or inhibitors of NADPH oxidases could alleviate cold tolerance (Zhou et al., 2012; Si et al., 2018).

Molecular and biochemical mechanisms supporting plant chilling and freezing tolerance are very complex and involve the activation of various genes in cell membrane metabolism, production of cryoprotective proteins and solutes like sucrose or proline, and production of ROS scavengers (Hannah et al., 2005; John et al., 2016). In *Arabidopsis*, low-temperature treatment modulates the expression of up to four thousand genes, and approximately half of these genes are upregulated and half down-regulated (Park et al., 2015; Zhao et al., 2016).

Expression analysis of mutant lines revealed that 10 - 20% of cold-modulated genes are regulated by C-repeat (CRT)-binding factors (CBFs), also known as dehydration responsive element (DRE)-binding factor1 (DREB1) proteins (Jia et al., 2016; Zhao et al., 2016). CBF/DREB1 is a subfamily of AP2/ERF DNA-binding transcription factors, and its members are involved in abiotic stress responses. Three members of this family (CBF1, CBF2, and CBF3) are well-known for their response to cold stress (reviewed in Shi et al., 2018). *CBF* expression is upstream regulated by the ICE1 transcription factor (Chinnusamy et al., 2003), and an increase in their expression is detectable already within minutes after cold exposure (Gilmour et al., 1998). Besides the transcriptional control, the CBF activity is regulated by post-translation modification *via* thioredoxin H2 in a temperature-dependent manner (Lee et al., 2021). The molecular analyses of *CBF* genes confirmed their positive effect on plant freezing tolerance accompanied by induction of downstream COLD-REGULATED (*COR*) genes (Gilmour et al., 2004; Jeknić et al., 2014).

A growing number of studies provide evidence that temperature and light signaling are closely connected, and light quantity and quality play a significant role in cold stress tolerance (Catalá et al., 2011; Jiang et al., 2017; Ahres et al., 2020; Prerostová et al., 2021a). Low light intensity was shown to decrease freezing tolerance in diverse plant species (Gray et al., 1997; Janda et al., 2007). However, the overall freezing tolerance depends probably also on the specific conditions for the cold acclimation, way of cultivation, and freezing treatment because plants can significantly increase freezing tolerance also under low light conditions, as we have recently reported (Novák et al., 2021). In addition to low temperatures, *CBF* genes are regulated by light quality, period, and circadian clocks (Franklin and Whitelam, 2007; Soitamo et al., 2008; Dong et al., 2011; Lee and Thomashow, 2012). At dusk and dawn, the ratio of red and far-red light (R/FR) decreases and positively regulates the expression of *CBFs* in *Arabidopsis* even at temperatures higher than those required for cold acclimation (Franklin and Whitelam, 2007). In tomato plants, a low R/FR light ratio increased the level of CBF transcript and overall cold tolerance of plants. The same effect of light was observed in *phyB* mutants but not in *phyA* mutants suggesting their diverse roles in response to cold (Wang et al., 2016). Phytochrome B was also shown to have a negative impact on cold tolerance in *Arabidopsis* (Franklin and Whitelam, 2007) and rice (He et al., 2016). On the contrary, a recent study shows PHYB to be a positive regulator of cold tolerance in tomato plants (Jiang et al., 2020). Recently, the blue light and photoreceptors cryptochromes were shown to play important role in cold tolerance and cold acclimation (Imai et al., 2021; Li et al., 2021a; Ma et al., 2021). These works confirmed the role of blue light in cold tolerance and suggested the presence of blue light-modulated cold tolerance mechanisms, including the activity of CRY2.

Besides the light, plant hormones also contribute to the cold stress response, and this is not limited only to well-known stress-related hormones like abscisic, salicylic, or jasmonic acid, but also cytokinin (reviewed in Jeon et al., 2010; Eremina et al., 2016; Prerostová et al., 2021b). Low temperature modulates cytokinin metabolism (Novák et al., 2021) and multiple signaling elements (Jeon et al., 2010; Zwack et al., 2016) to adjust freezing tolerance. Recently, the role of strigolactones in cold response was also discovered (Zhang et al., 2020).

The model species *Arabidopsis thaliana* is an annual plant with a wide geographical distribution. *Arabidopsis* originates in Eurasia, and its occurrence extends from North Africa to northern Europe through eastern and central Asia and North America. Its natural habitats thus include areas that have very different local climates and different winter temperatures (Hoffmann, 2002; Koornneef et al., 2004). The natural variation of *Arabidopsis* accessions provides an excellent tool for exploring the molecular basis of adaptation. It was reported that ecotypes differ in their tolerance against abiotic stresses, including cold, with those coming from colder northern latitudes being more tolerant to frost than those from southern areas (Hannah et al., 2006; Gery et al., 2011; Gehan et al., 2015).

Previously, we have shown that *Arabidopsis* plants could be hardened even at low white light intensity (Novák et al., 2021). However, there is limited knowledge about the interaction between low temperature and light quality. In particular, the comparison of the effect of red and blue light was not studied in detail. We have assessed the quantity of blue and red light in our previous cultivation setup and treated plants with low temperature and specific light quality and intensity. We show that blue light promotes the effect of the low temperature on the level of *Arabidopsis* physiology parameters, gene expression, and proteome. Our experiments with selected contrasting *Arabidopsis* accessions revealed a correlation of accession latitude with cold-induced ion leakage and accession-specific cold response proteins. Furthermore, our data indicate that light quality played a regulatory role in response to low temperatures. We showed that cold treatment under red light attenuated oxidative stress and promoted photosynthesis and that blue light cold-treated plants upregulated stress marker genes, the key cold-response regulatory genes CBFs, and accumulated a higher level of cold response proteins. Finally, the plant freezing tolerance assay suggested the importance of complementarity of both light-quality modulated responses.

## Materials and Methods

### Plant Material and Growth Conditions

*Arabidopsis thaliana* accessions Colombia (Col-0), RRS (RRS-7 and RRS-10), Wassilewskija (Ws-0 and Ws-2), Tossa de Mar (Ts-1), and Tammisari (Tamm-2) were grown in a hydroponic system (Araponics, Liege, Belgium). Seeds were surface-sterilized with 70% EtOH and 50% bleach with 0.05% Triton X-100. Then, the seeds were stratified for 2 days at 4°C in the dark and sown on 0.7% (w/v) agar in seed holders immersed in regularly refreshed ½ Murashige and Skoog (MS) medium. The germinating plants were cultivated under short-day conditions (8 h light) in an AR-36L growth chamber (Percival Scientific Inc, Perry, IA, United States) with 65% relative humidity and 21/19°C day/night temperatures with 100 μmol m^−2^ s^−1^ PPFD provided by Philips TL-D fluorescent tubes. Plants aged 5 weeks were divided into five groups (*n* > 30) and were cold-treated at 4°C for 7 days in short-day conditions under (i) standard light intensity and white light (W100), (ii) low light intensity and white light (W20), (iii) low light intensity of blue light (430 nm; B20), or (iv) low light intensity of red light (660 nm; R20). The control group of plants continued to grow at 21 /19°C and standard white light intensity (100 μmol m^−2^ s^−1^); 20 and 100 μmol m^−2^ s^−1^ PPFD were chosen as the ‘low' and ‘standard' light intensities for reasons outlined in the Discussion. The photoperiod and humidity were the same in all treatments. After this period, young leaves from at least six plants per sample and experimental replicate were collected, flash-frozen in liquid nitrogen, homogenized, and aliquoted for the molecular analyses. The whole experimental design is visualized in Supplementary Material S1.

For freezing experiments, *Arabidopsis thaliana* plants (Col-0) were cultivated *in vitro* on Petri dishes on 0.8% ½ MS medium at short-day conditions (8/16 day/night) and 21°C/19°C day/night temperatures with 100 μmol m^−2^ s^−1^ PPFD during the day. After 2 weeks of horizontal cultivation, the plants were transferred to low temperature (4°C) for 1 week at different light conditions: W100, W20, B20, and R20. At this point, plants reached the growth stage 1.04 (Boyes et al., 2001). The freezing treatment was performed in dark conditions. The cold-treated plants and 2-weeks-old control plants without cold treatment were exposed to a decreasing temperature at a rate of 2°C per hour until it reached−6°C and at this point, ice nucleation was induced. Then, the temperature was reduced to a final−7°C. After 2 h of exposure to−7°C, the plates with plants were removed and allowed to thaw at 4°C. The freezing tolerance was evaluated after 14 days of recovery at standard conditions (21°C/19°C day/night temperatures, 100 μmol m^−2^ s^−1^ PPFD). The freezing tolerance rating was determined by counting the number of plants which continued growth, reaching at least the growth stage 1.06. Plants which did not match this criterion (dead or growth-arrested) were considered freezing intolerant.

### Evaluation of Plant Physiology

The plants' growth at both the 1.14 growth stage (start of the experiment) and 7 days later (the end of the cold treatment at 4 and 21°C control treatments) was evaluated by quantifying rosette dimensions using ImageJ software (Schneider et al., 2012). The average shoot fresh weight and dry weight of plants subjected to each treatment were evaluated at the end of the cold period. Chlorophyll fluorescence measurements were performed using a kinetic imaging fluorometer (Handy FluorCam, model FC 1000-H, Photon Systems Instruments; www.psi.cz) as previously described (Novák et al., 2021). Anthocyanins were determined according to Zhang et al. (2011) with the following modifications: 0.08 g of leaf tissue was extracted overnight in 0.45 ml of 1% (v/v) hydrochloric acid in methanol, then chlorophyll was removed by partitioning into a chloroform fraction formed by adding 0.15 ml chloroform and 0.30 ml water. Spectrophotometrically determined levels of pigments were normalized to dry weight. The extent of lipid peroxidation was determined by estimating their malondialdehyde content (MDA) according to Novák et al. (2013). The membrane damage was determined by electrolyte leakage measurement. Whole-leaf rosettes (5-6 for each variant) were incubated in 0.01% Silwet solution (Crompton) for 1.5 h at RT with gentle shaking. The initial electrolyte leakage of samples (EL_in_) and blank (EL_B1_) was measured by electrical conductivity. To release all electrolytes, the samples were boiled for 0.5 h at 100°C and after cooling down to RT, the conductivity of the solution was measured again for samples (EL_fin_) and blank (EL_B2_). The electrolyte leakage was calculated using the formula: EL (%) = [(EL_in_- EL_B1_)/ (EL_fin_- EL_B2_) × 100].

### RT-qPCR Analysis

The total mRNA was extracted from portions of the samples according to Valledor et al. (2014) with some modifications (Supplementary Material S2). RT-qPCR was performed as described by Novák et al. (2015), using primers listed in Supplementary Material S3. Three biological replicates of each variant were analyzed. The relative levels of gene expression were normalized by the delta-delta Ct method (Pfaffl, 2001), using *ACT2*, and *EF1-*α as the reference genes.

### Proteomic Analysis

Total protein extracts were prepared as described by Valledor et al. (2014), then portions of samples corresponding to 5 μg peptide were analyzed by nanoflow reverse-phase liquid chromatography-mass spectrometry using a 15 cm C18 Zorbax column (Agilent), a Dionex Ultimate 3000 RSLC nano-UPLC system and Orbitrap Fusion Lumos Tribrid Mass Spectrometer (Thermo Fisher). Acquired spectra were recalibrated and searched against the Araport 11 protein database following Berková et al. (2020). Only proteins represented by at least two unique peptides were considered for the quantitative analysis. The quantitative differences were determined by Minora, employing precursor ion quantification followed by normalization and calculation of relative protein abundances. The mass spectrometry proteomics dataset has been deposited in the ProteomeXchange Consortium database (Vizcaíno et al., 2016) *via* the PRIDE partner repository with the identifier PXD033102.

### Statistical Analysis

Statistical tests were implemented using Instant Clue (Nolte et al., 2018), Rapid Miner (www.rapidminer.com; Mierswa et al., 2006), R (R Development Core Team, www.r-project.org), MetaboAnalyst 5.0 (Chong et al., 2019), SIMCA 14.1, Proteome Discoverer 2.4, Compound Discoverer 3.2, and Statistica 12 software packages. The detected differences between samples were deemed significant if *p* < 0.05.

## Results

### Light Intensity and Spectral Quality Affect Response to the Low Temperature of *Arabidopsis thaliana* (Col-0)

**Figure 1:**
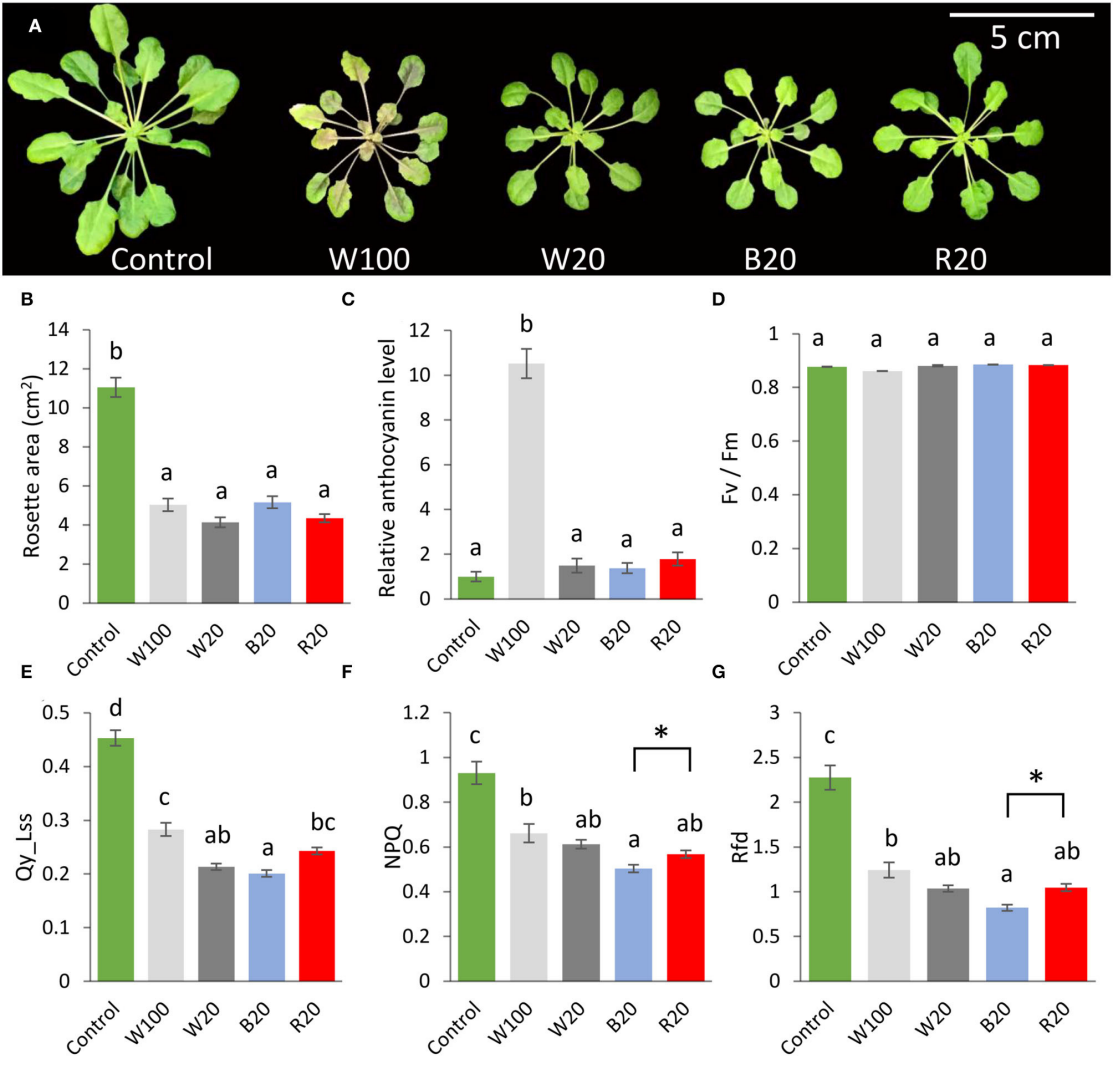
Light intensity and quality modulate *Arabidopsis* response to low temperature. **(A)** Representative images of *Arabidopsis* Col-0 plants grown for 7 days in indicated conditions. **(B)** Rosette area. **(C)** Relative anthocyanin contents normalized to dry weight (relative to non-treated control plants). **(D)** The maximum quantum efficiency of photosystem II in the dark-adapted state. **(E)** Quantum efficiency of photosystem II in light steady state. **(F)** Non-photochemical quenching in light steady state. **(G)** Photosynthetic parameter Rfd (fluorescence decline ratio in light steady state). Results are means of three biological replicates, error bars indicate standard errors, and letters in all panels indicate significant differences according to one-way ANOVA with Tukey's HSD *post hoc* test (*p* < 0.05). Asterisks indicate significant differences according to Student's *t*-test between marked variants (*p* < 0.05). W100, cold treatment under 100 μmol m^−2^ s^−1^ white light; W20, cold treatment under 20 μmol m^−2^ s^−1^ white light; B20, cold treatment under 20 μmol m^−2^ s^−1^ blue light; R20, cold treatment under 20 μmol m^−2^ s^−1^ red light. For details, see Materials and methods.

The 5-week-old *Arabidopsis thaliana* (Col-0) plants grown in a hydroponic system were used to elucidate the effects of light quality in low-temperature treatment (Figure 1A). The plants were cold-treated at 4°C for 7 days in short-day conditions at (i) standard light intensity and white light (W100), (ii) decreased light intensity and white light (W20), (iii) decreased light intensity of blue light (B20), or (iv) decreased light intensity of red light (R20). The leaves of cold-treated plants showed a similar pattern at the end of the cold treatment but were significantly inhibited in growth compared to non-treated plants grown at short-day conditions with standard white light intensity and 21°C (control; Figure 1B). The cold-treated W100 plants had darker leaf coloration reflected in higher anthocyanin accumulation compared to cold-treated plants grown at low light intensity (under white, blue, or red light; Figure 1C).

The analysis of chlorophyll fluorescence did not confirm a significant change in maximum efficiency of photosystem II (Fv/Fm ratio) in the cold-treated plants (Figure 1D), but cold stress was followed by a significant decrease in efficiency of photosystem II in a light steady state (Qy_Lss; Figure 1E). The most significant inhibition was observed in plants grown under blue light with a more than 50% decrease in this parameter compared to non-treated control plants. Similarly, non-photochemical quenching (NPQ, which reflects protective mechanisms of energy dissipation by heat) and vitality index (Rfd) were reduced in cold-treated plants with the most significant change in blue light-grown plants (Figures 1F,G).

Physiological experiments have shown that the cold response of *Arabidopsis* is affected by both light intensity and spectral properties of light, and photosynthetic parameters suggested a significant role of blue light in response to low temperature.

### Cold-Treated Plants Under Standard White Light and Low Blue Light Show Increased Cold Stress Markers

**Figure 2:**
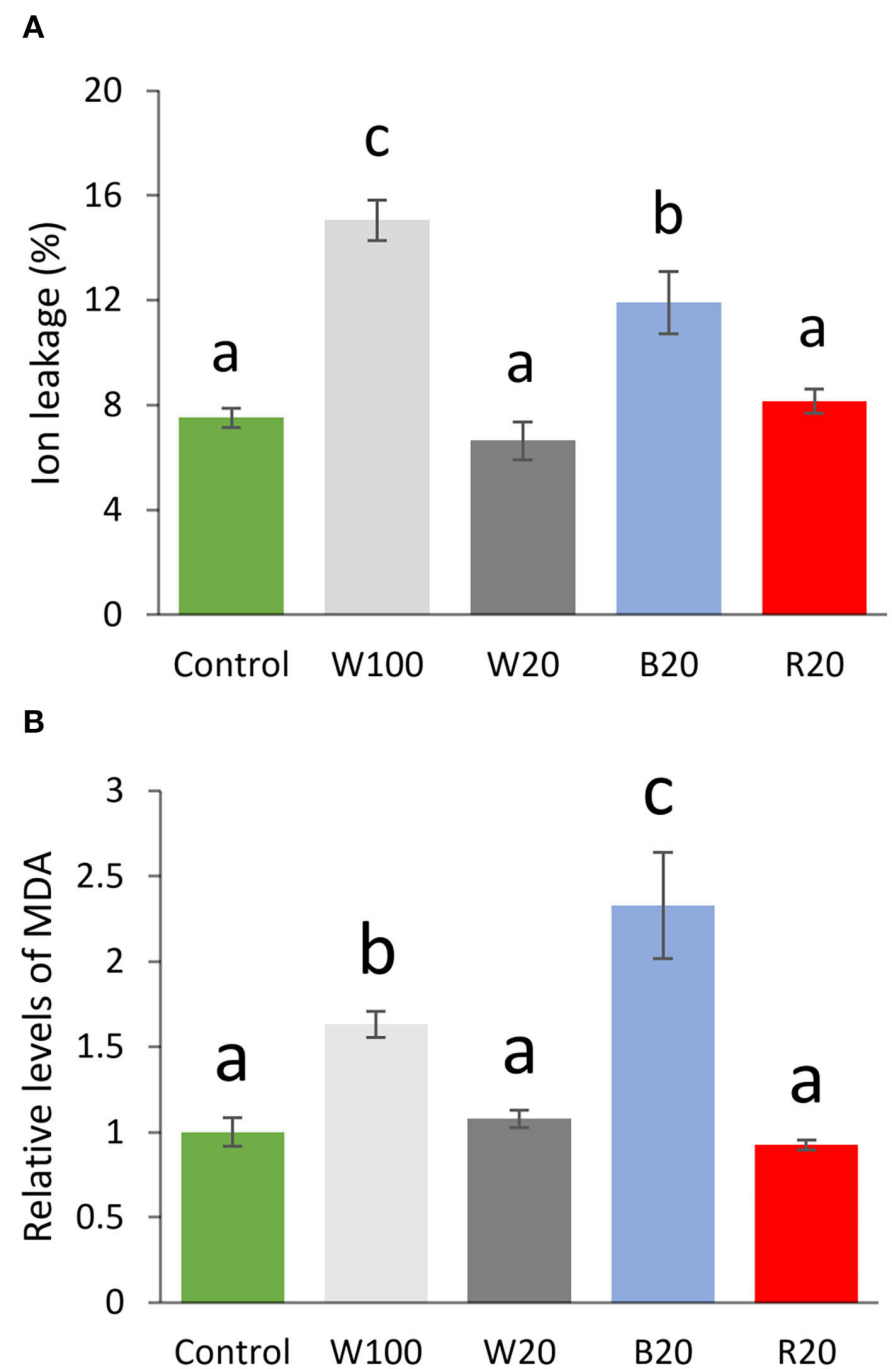
Membrane damage and lipid peroxidation of plants treated by low temperature under different light regimes. **(A)** Ion leakage and **(B)** relative level of malondialdehyde (MDA) normalized to control and compared to control. Results represent means of three biological replicates with standard errors, letters indicate significant differences (ANOVA, Tukey's HSD *post hoc* test, *p* < 0.05). W100, cold treatment under 100 μmol m^−2^ s^−1^ white light; W20, cold treatment under 20 μmol m^−2^ s^−1^ white light; B20, cold treatment under 20 μmol m^−2^ s^−1^ blue light; R20, cold treatment under 20 μmol m^−2^ s^−1^ red light. For details, see Materials and methods.

The role of light quantity and quality in cold response was evaluated by ion leakage measurement. Results did not show any difference among W20, R20, and control plants but a significant (*p* < 0.05) increase in ion leakage for W100 and B20 cold-treated plants (Figure 2A). The relative content of malondialdehyde, the product of lipid peroxidation, confirmed that only W100 and B20 plants showed a response to oxidative stress higher than the control plants (Figure 2B). Interestingly, the comparison of B20 and R20 plants showed that B20 plants had a more than 45% higher ion leakage and 130% higher MDA level than R20 plants.

### Cold-Treated Plants Under Blue and Red Light Showed Reduced Tolerance to Freezing

**Figure 3:**
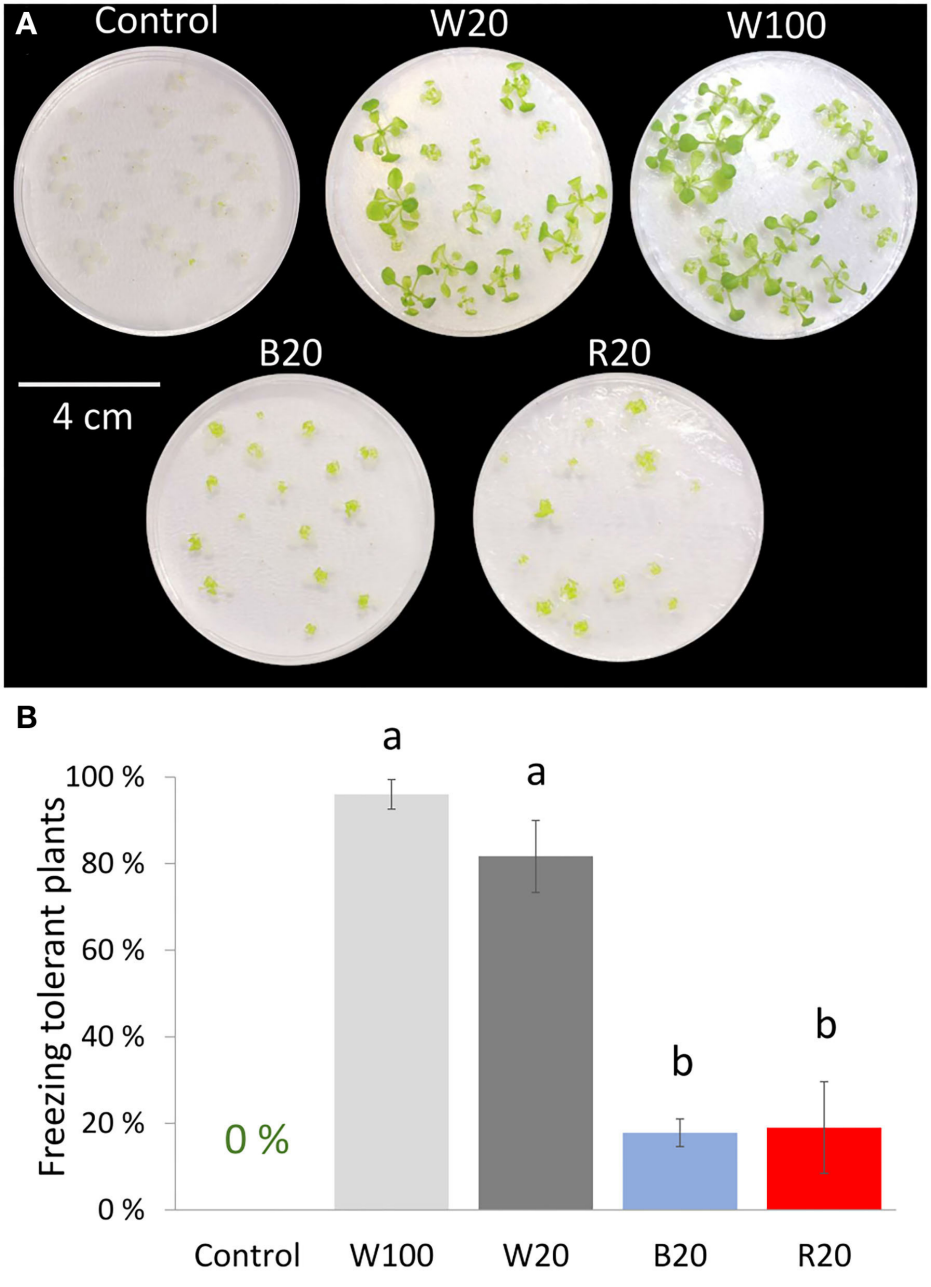
Freezing tolerance of cold-treated plants under different light regimes. **(A)** Representative images of *Arabidopsis* Col-0 plants 2 weeks after freezing stress; 2-week-old plants cultivated on Petri dishes in standard conditions were treated at low temperature (4°C) under different light conditions for a week (W100, W20, B20, R20) and then exposed to freezing stress. Control plants represent 2-week-old plants exposed to freezing stress without the cold treatment. Plants were transferred to control conditions after the freezing stress and recovered for another 2 weeks. **(B)** Freezing tolerant plants. Plants that produced new leaves were evaluated as freezing tolerant. Results represent means of three biological replicates with standard errors, letters indicate significant differences (ANOVA, Tukey's HSD *post hoc* test, *p* < 0.05). For details, see Materials and methods.

The role of light conditions in the acquired freezing tolerance of plants was tested. For this validation experiment, a modified experimental setup was utilized, employing 2-week-old plants grown on Petri dishes and then exposed to 4°C under W100, W20, B20, or R20 light conditions for a week. The cold treatment period was followed by a freezing stress experiment confirming the positive role of low-temperature treatment on plant freezing tolerance. In contrast to cold-treated plants, control plants (3-week-old plants without cold treatment) were not able to survive freezing treatment. However, there were significant differences among the cold-treated light variants. Cold-treated plants under standard light conditions W100 showed the highest freezing tolerance (Figures 3A,B). Cold treatment under low light intensity was very sensitive to the light spectrum. W20 cold-treated plants acquired similar tolerance to freezing as W100 plants, but B20 and R20 cold-treated plants showed very limited tolerance to freezing stress.

### Blue Light Amplifies the Effect of Low Temperature in Diverse *Arabidopsis* Accessions

*Arabidopsis* is a widespread plant species, and its accessions had to adapt to their location-specific temperature profiles and light conditions. Previous studies have shown a correlation between the cold response of *Arabidopsis* accessions and latitude or temperature in the original growth habitat (Zuther et al., 2012). The light intensity and spectral properties of sunlight vary across latitudes over the year, therefore, we have tested the specific role of light quality in cold response in another six accessions ranging between 40 and 60°, and adapted to different average temperatures (Supplementary Material S4).

**Figure 4:**
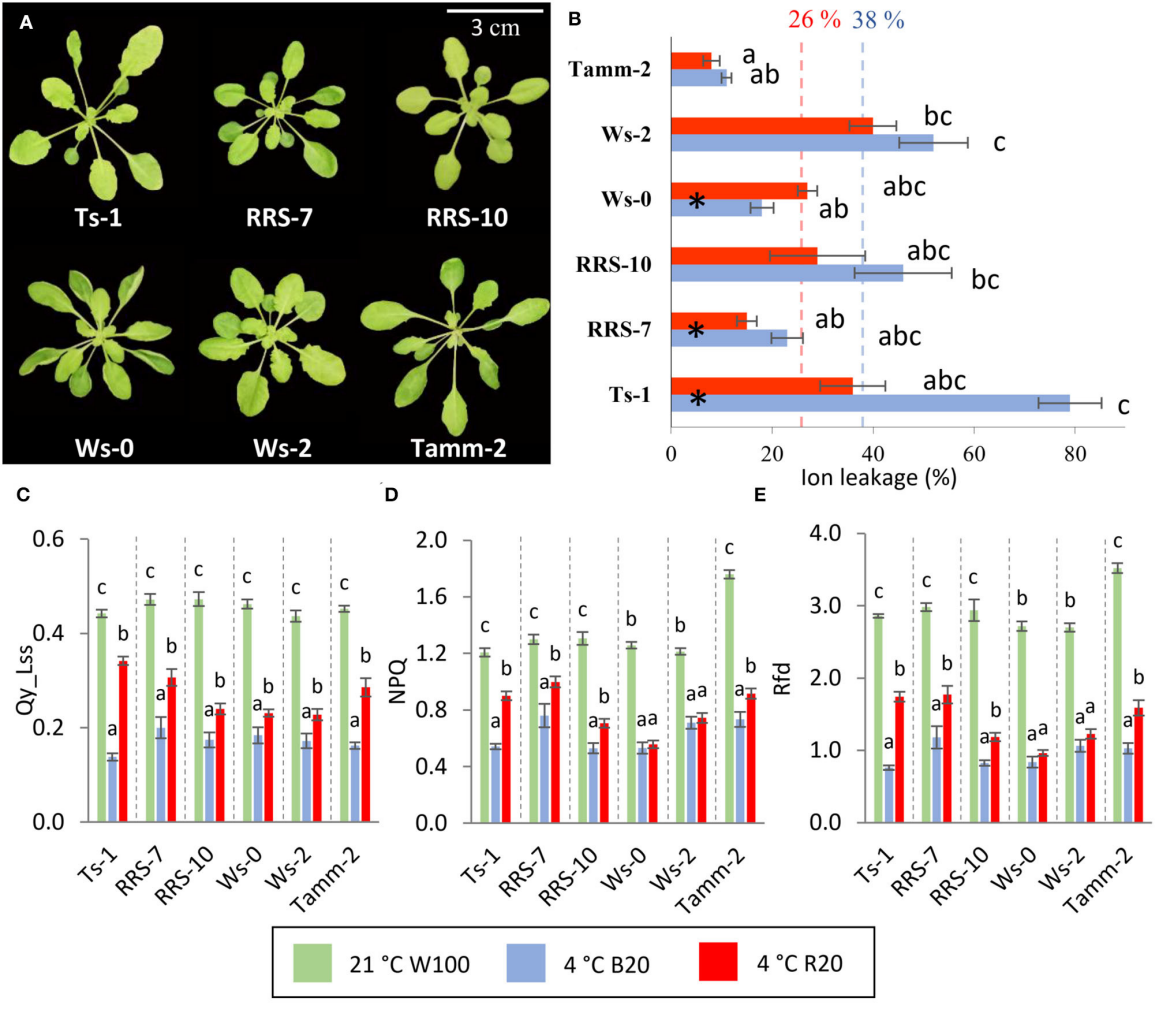
Role of blue and red light in the cold-treated *Arabidopsis* accessions. **(A)** Representative images of 5 weeks old hydroponically grown *Arabidopsis* accessions grown in control conditions and used for cold treatment experiments. Plants were treated for 7 days at 4°C under 20 μmol m^−2^ s^−1^ blue or red light. **(B)** Ion leakage of cold-treated plants in respective conditions. Results are means of three biological replicates, error bars indicate standard errors, and letters indicate significant differences according to the Kruskal–Walli's test with *post hoc* analysis (*p* < 0.05). Black asterisks in columns indicate significant differences between red and blue light cold-treated accession according to Student's *t*-test (*p* < 0.05). Colored dashed lines represent the average value of ion leakage for blue and red light cold-treated plants. **(C)** Quantum efficiency of photosystem II in light steady state, **(D)** non-photochemical quenching in light steady state, **(E)** photosynthetic parameter RFD. Results of photosynthetic parameters are means of three biological replicates, error bars indicate standard errors. The statistical significance of the results was evaluated separately for each accession. Letters indicate significant differences according to one-way ANOVA with Tukey's HSD *post hoc* test (*p* < 0.05). Accessions Ts-1, RRS-7, RRS-10, Ws-0, Ws-2, and Tamm-2 are described in Supplementary Material S4.

All accessions were grown for 5 weeks in a hydroponic system in short-day conditions with a standard light photon flux density of 100 μmol m^−2^ s^−1^ before cold hardening. At this growth stage, different accessions showed diverse rosette and leaf shapes (Figure 4A). Most significant was a reduction of rosette compactness of Ts-1 and Tamm-2.

Next, plants were cold-treated under low temperature (4°C) and blue or red light with a photon flux density of 20 μmol m^−2^ s^−1^. At the end of the cold treatment period, ion leakage was determined (Figure 4B). All accession except Ws-0 showed higher ion leakage if cold-treated under blue light. Ion leakage of blue light cold-treated plants correlated statistically significantly (*p*<0,05) to the average (January to March) temperature of the original location of accession (Supplementary Material S4) and was on average almost 50% higher compared to red light (Figure 4B). The ion leakage of B20 plants also negatively correlated with the number of ground frost days and latitude of the original location (Supplementary Material S5). The ion leakage of R20 plants correlated with the temperature of the original location to a lower extent, and this correlation was not statistically significant. The main factor reducing correlation was probably a high decrease in ion leakage of red light cold-treated Ts-1 accession.

The analysis of photosynthetic parameters confirmed the blue light as an amplifier of the low-temperature treatment. The determination of Qy_Lss showed more than 40 and 60% decrease in cold-treated plants under red and blue light respectively (Figure 4C). The most significant change between blue and red light-treated accession was found in Ts-1. Interestingly, Qy_Lss in cold-treated plants under blue and red light showed an inverse correlation with the temperature of the original location of accession (Supplementary Material S5). NPQ was also more significantly affected by the blue light during cold treatment but not in all accessions (Figure 4D). Ws-0 and Ws-2 did not show a significant difference between blue and red light. The highest NPQ was measured in northern accession Tamm-2 and accession RRS-7. Vitality parameter Rfd showed a similar pattern to Qy_Lss, a higher reduction in plants treated by low temperature under blue light, and the highest impact of light in accession Ts-1 (Figure 4E).

Physiological experiments employing diverse *Arabidopsis* accessions supported the amplifying role of blue light in low-temperature response and confirmed its general activity across accessions. Statistical analysis showed the correlation of cold-induced membrane damage with physical parameters of accession habitats.

### Different Light Quality During Cold Treatment Does Not Change the Expression Profile of Cytokinin-Related Genes But Plays a Significant Role in the Expression of CBF Genes

**Figure 5:**
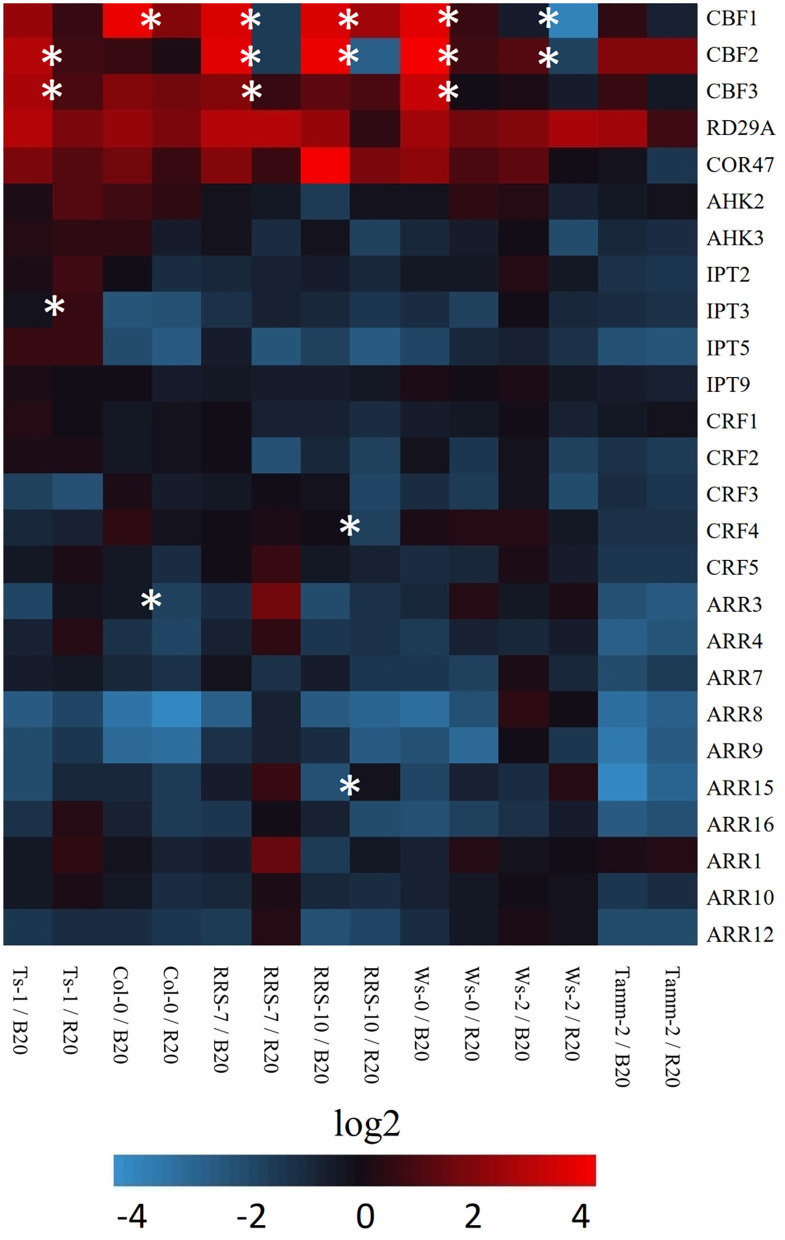
Heat map visualization of expression of selected cold-related and cytokinin-related genes. The expression was measured in leaves of *Arabidopsis* accessions treated 7 days by low temperature under red (R20, 20 μmol m^−2^ s^−1^) or blue light (B20, 20 μmol m^−2^ s^−1^) conditions by RT-qPCR. Data were normalized as specified in Material and methods. Results are means of three biological replicates, and asterisks indicate significant differences between red and blue light-grown accession according to Student's *t*-test (*p* < 0.05). Accessions Ts-1, RRS-7, RRS-10, Ws-0, Ws-2, and Tamm-2 are described in Supplementary Material S4.

The CBF signaling pathway regulates essential aspects of cold response and cold tolerance. CBF genes are activated within minutes following cold treatment and stimulate downstream targets known as CBF regulon. The role of light quality in the expression of CBF genes and cold-related genes was analyzed by RT-qPCR (Figure 5). The analysis confirmed the importance of light quality in the regulation of CBF expression. Plants treated by low temperature under blue light have significantly increased expression of CBF genes in comparison to red light cold-treated plants. The exception was northern accession Tamm-2 and southern accession Ts-1 with the expression of CBF independent of light quality. Interestingly, light-dependent expression was not significant for two analyzed COR genes *RD29A* and *COR47*.

The recently published works highlighted the potential role of cytokinin in cold response. The analysis of expression of cytokinin-related genes at the end of the cold treatment period did not confirm differences in response to cold under blue or red light (Figure 5). The analysis confirmed that the expression of cytokinin transcription factors CRF and ARR-B was predominantly down-regulated. Similarly, the cytokinin response genes ARR-A were down-regulated (Figure 5). Out of seven different accessions, only Ts-1 showed statistically significant differences in the expression of cytokinin biosynthetic enzyme, namely *IPT3*.

### Light Quality Modulated Cold-Stress Responsive Proteome and the Response Was Predominantly Accession-Specific

**Figure 6:**
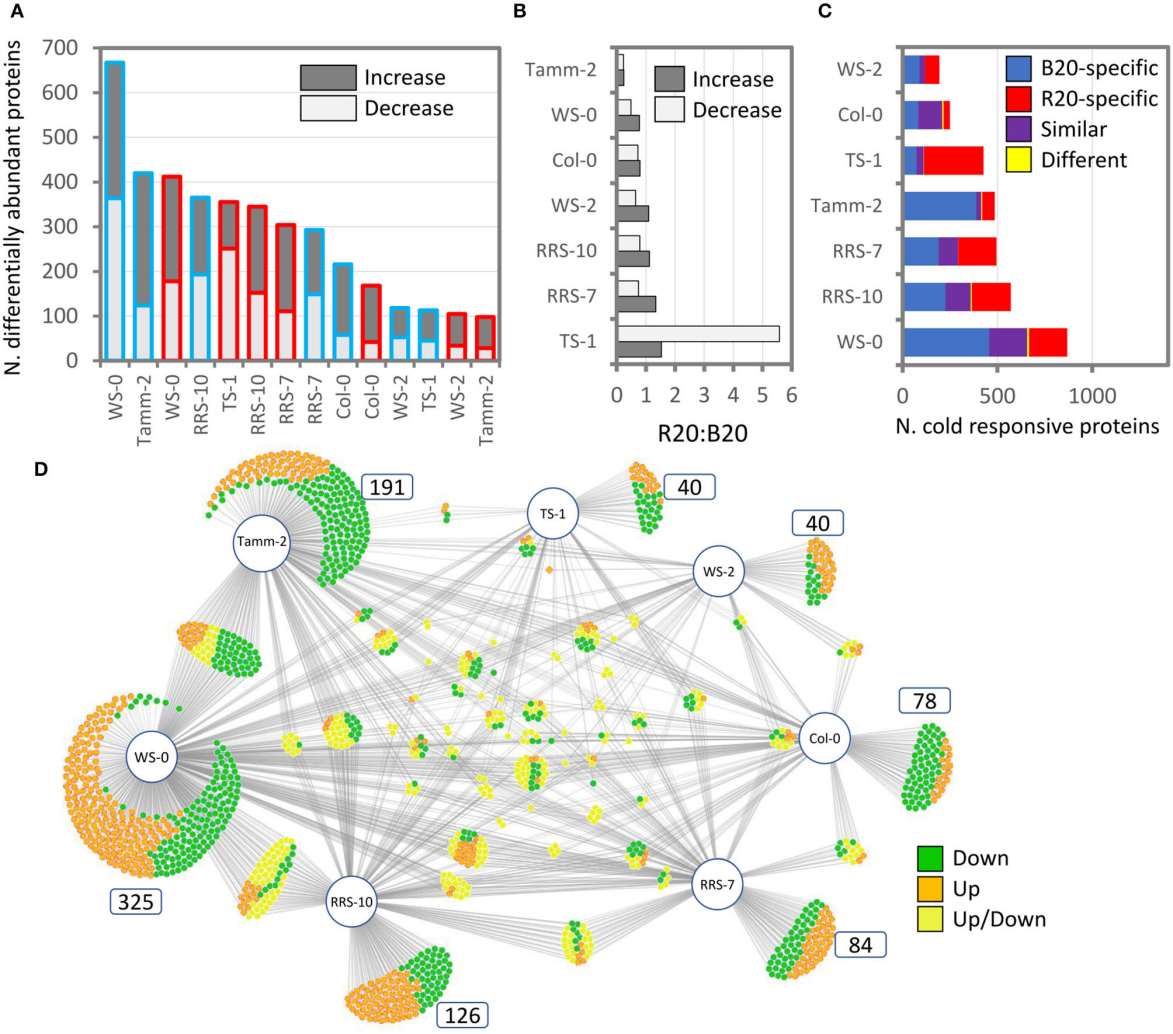
Comparison of cold stress response in *Arabidopsis* accessions. **(A)** Differentially abundant proteins in leaf proteomes of plants grown under red (R20; red outline) or blue (B20; blue outline) light at 4°C for 7 days compared to respective accessions grown under standard conditions (21°C, W100). **(B)** Comparison of ratios between the number of cold-responsive proteins in R20 vs. B20 plants. **(C)** Cold stress in R20 and B20 plants elicits unique sets of proteins. **(D)** The cold response is accession-specific. Comparison of response to cold stress in B20 plants visualized with DiVenn 2.0 (Sun et al., 2019). For details, see Supplementary Material S6.

The analysis of shoot proteome of *Arabidopsis* accessions provided identification and quantitative data for 3,124 and 2,290 *Arabidopsis* proteins, respectively. The effect of cold stress was found to be both light-quality- and accession-dependent (Figures 6A–D). The lowest response to cold treatment was found in the accession Ws-2 with only 105 (R20) and 118 (B20) differentially abundant proteins compared to the plants grown under control conditions (Figure 6A). In contrast, the abundances of over 600 proteins were significantly altered in B20 Ws-0 plants. In most accessions, B20 plants showed a higher response to cold stress, with more cold-responsive proteins with an increase in abundance and fewer proteins with a decrease in abundance than the corresponding R20 plants (Figure 6B). The comparison of B20 and R20 plants revealed only limited overlap, but the majority of R20 and B20 responsive proteins showed a similar response to cold stress (Figure 6C). Interestingly, Col-0 plants showed the second weakest response to cold, and the similarity between R20 and B20 plants was the highest for this accession with more than 50% of shared cold-responsive proteins. As illustrated in the B20 cold response, most cold-responsive proteins were accession-specific (Figure 6D). The accession-specific differentially abundant proteins represented 60 and 66% of all differentially abundant proteins in B20 and R20 plants, respectively.

### Identification of Proteins With a Putative Role in Contrasting Cold Responses in R20 and B20 Plants

**Figure 7:**
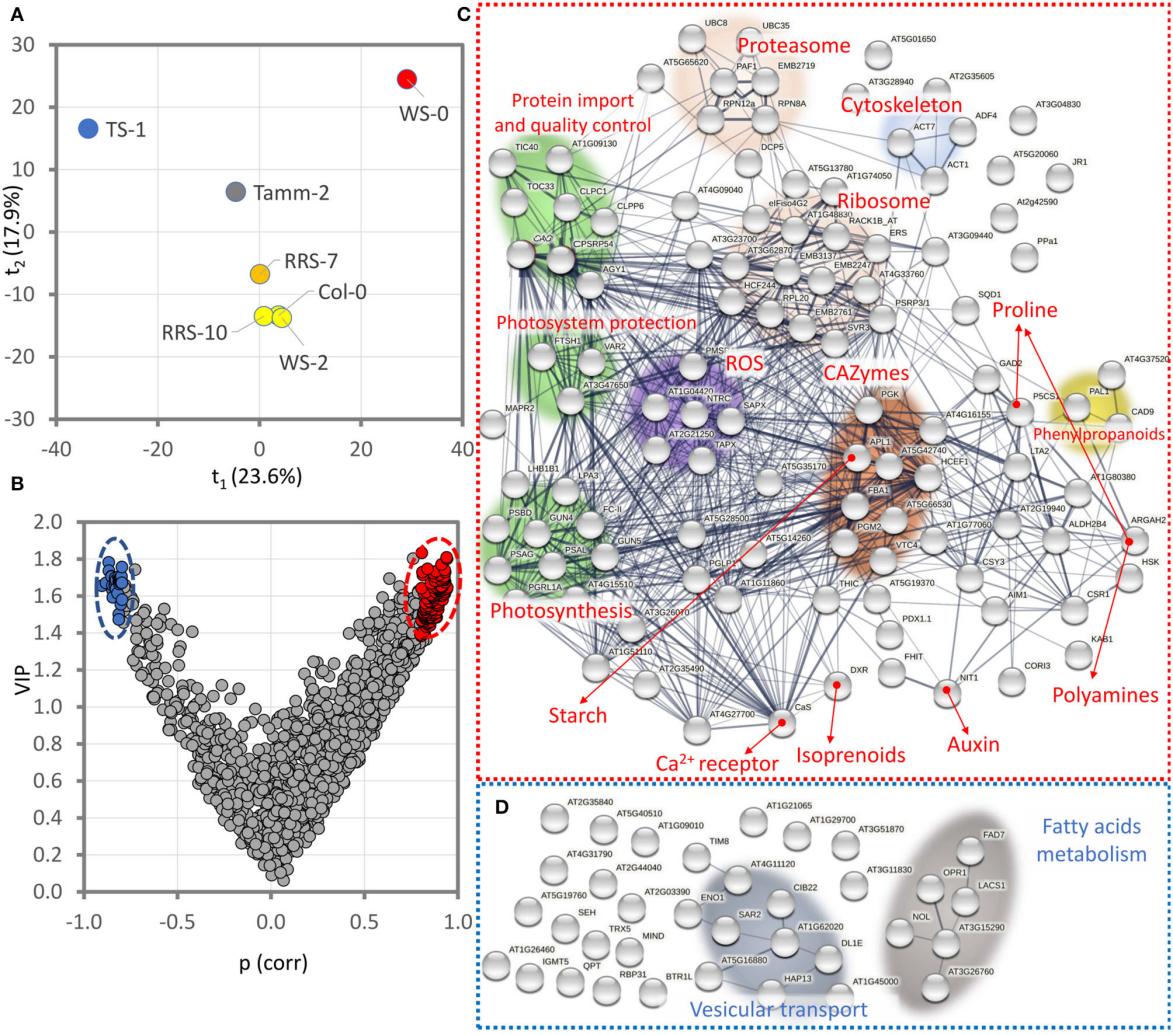
Identification of putative protein markers of the contrasting response in R20 and B20 plants under cold stress. The ratios in protein abundance, ion leakage, Qy_lss, NPQ, and RFD were analyzed by partial least squares regression analysis **(A)** and VIP variables of importance in projection. **(B)** Projected ratios represent the means of at least four biological replicates. **(C,D)** Functional characterization of identified proteins showing positive correlations [red, *p*(corr)>0.8, VIP>1.4] and negative correlations [blue, *p*(corr)<-0.8, VIP>1.4] with the OPLS distribution. Visualized by STRING (Szklarczyk et al., 2021). For details, see Supplementary Material S7.

Proteomics data supported significant differences between R20 and B20 plants under cold stress. To identify putative protein markers contributing to the observed differences in ion leakage and photosynthetic parameters, ratios between R20 and B20 protein abundances were analyzed by partial least squares regression analysis (PLS; Figures 7A–D). The analysis separated accessions Ts-1 and Ws-0 (Figure 7A), and the consecutive VIP (variables of importance in projection) highlighted 108 and 35 proteins that showed positive and negative correlations with the observed separation, respectively (Figure 7B). Proteins that were found to be more abundant in R20 plants (Figure 7C) were predominantly localized in chloroplasts (58%), indicating that this organelle is critical for the observed differences between R20 and B20 response to cold stress. The functional characterization of these proteins highlighted an increase in the components of photosystems, protein import into chloroplasts, and mechanisms involved in plastidic protein repair and quality control. A higher abundance was also found for carbohydrate-active enzymes (CAZymes), proteins of ROS metabolism and signaling, components of proteasome, translation machinery, cytoskeleton, and phenylpropanoid pathway. Proteins correlating with the observed B20-promoted cold stress response were enriched only in two categories, namely vesicular transport and fatty acid metabolism (Figure 7D).

## Discussion

### Role of Light Quality and Quantity in Response to Low Temperature

Our previous study showed that *Arabidopsis* plants exposed to low temperature under a reduced white light intensity of 20 μmol m^−2^ s^−1^ could increase their resistance to freezing (e.g., Novák et al., 2021; Prerostová et al., 2021a). However, the role of light quality was not analyzed in detail. Other groups have reported and highlighted the impact of the blue and red light spectrum, or R/FR light ratio in plant response to low temperature (Franklin and Whitelam, 2007; Imai et al., 2021). But the overall effect of red and blue light on plants treated with low temperature was not compared. Here, to further deepen our knowledge about the role of the light spectrum in cold response, month-old plants grown under 100 μmol m^−2^ s^−1^ white light were exposed for 7 days to a low non-freezing temperature under 20 μmol m^−2^ s^−1^ of red or blue light. The selected photon flux density of 20 μmol m^−2^ s^−1^ was matching the corresponding photon flux density of blue and red components in 100 μmol m^−2^ s^−1^ white light fluorescent tube sources. This experimental design resolved the specific effect of individual light regions and separated the integrative net effect of remaining white light components. Moreover, these light intensities correspond to natural conditions that plants encounter in winter (Robson and Aphalo, 2019). Furthermore, photoreceptors of blue and red light are sufficiently activated at this intensity (Park et al., 2012; Liu et al., 2020b), and reduced temperature prolongs their activated state (Legris et al., 2016; Pooam et al., 2021). The action spectrum for photosynthesis is comparable for the blue and red light components, and approximately the same amount of energy was available for photosynthesis in our experiment. Thus, it is expected that the observed changes in physiology and molecular processes are predominantly proportional to the differences in light perception and signaling.

One of the well-known effects of cold stress is the inhibition of plant growth. In accordance, there was no statistically significant difference in the rosette size R20, B20, W20, and W100 plants after 7 days of cold stress (Figure 1B). It has been reported that the inhibitory effect of suboptimal temperature can be compensated by red light, and plants grown under red light at 16°C had a much larger leaf rosette compared to the variant under blue light (Liu et al., 2020a). Results reported here indicate that the effect of temperature (4°C) on growth overcomes the impact of light quality. Growth inhibition could be the result of metabolic activity attenuation and seems to be critical for cold resilience. Indeed, activation of growth signals under low temperature has a negative impact on plants chilling tolerance (Lange et al., 2020; Li et al., 2021b).

### Cold-Induced Anthocyanin Accumulation Is Lost in R20 and B20 Plants

The effect of reduced temperature is associated with reprogramming of metabolism and activation of pathways for the synthesis of protective compounds, including anthocyanins (Novák et al., 2021). These cold-responsive compounds may contribute to plant resistance to low temperatures (Catalá et al., 2011). A significant accumulation of anthocyanins was detectable only in plants grown under white light (at optimal light intensity; Figure 1C). At normal temperatures, the accumulation of anthocyanins is more sensitive to blue light than red light (Liu et al., 2018b), and significant differences can be detected at low light intensities (Vandenbussche et al., 2007). That indicates that the light-induced anthocyanin biosynthesis regulation is temperature-dependent, requiring sufficient light intensity.

### Blue and Red Light Cold-Treated Plants Showed Similar Freezing Tolerance But Different Level of Cold-Induced Stress Markers

The comparison of B20 and R20 plants showed significant differences in ion leakage and photosynthetic parameters (Figures 1D–G, 2A). The observed significant increase in ion leakage and lower value of RFD indicated higher oxidative stress in B20 plants, and that was confirmed by measuring the product of membrane oxidation MDA (Figure 2B). Reactive oxygen species (ROS) play a significant role in cold acclimation and their levels are increased in response to cold stress (Li et al., 2014; Zhang et al., 2020). It is well-known that ROS accumulation is harmful to the plant (e.g., Novák et al., 2013; Saxena et al., 2016), but recent reports indicate that ROS participate in the ICE-CBF pathway regulation and may (at a certain level) serve as signaling molecules and increase freezing tolerance (Wang et al., 2018; Devireddy et al., 2021; Fang et al., 2021). In addition, it has been shown that the activity of the CBF transcription factors is regulated by redox-mediated switching by TRX-H2, the thioredoxin translocated from cytoplasm to nucleus in response to low temperature (Lee et al., 2021). Our proteomics data indicated that two proteins of the TRX-H family were significantly less abundant in some B20 and R20 accessions (TRX-H3, TRX-H5). However, these changes did not seem to correlate with the freezing tolerance (Table S5). Interestingly, thioredoxin reductases were implicated in the contrasting cold responses in R20 and B20, namely NTR3 and FTRC (Figure 7C, Supplementary Material S6). That is well in line with the previously reported role of cellular redox status in an optimal cold response (Lee et al., 2021).

A higher ROS accumulation in B20 plants may coincide with the cold-regulated cryptochrome activity (Pooam et al., 2021). Cryptochromes positively affect ROS in the cell nuclei as a part of the signaling cascade (Consentino et al., 2015). This production does not represent a significant part of the ROS production at normal temperature (El-Esawi et al., 2017), but it is possible that its importance increases with the cold-induced inhibition of photosynthetic light reactions (Figures 1E–G). This may be another factor in amplifying the cold response in the blue light cold-treated plants.

Increased stress markers did not correlate with plant freezing tolerance (Figure 3). Similar to Li et al. (2021a), plants exposed to low temperatures under white light showed the highest freezing tolerance. High freezing tolerance in W20 cold-treated plants and reduced tolerance in B20 and R20 plants suggest a higher impact of light quality than light quantity in the cold response and cold acclimation process.

### Accession-Specific Responses May Reflect *Arabidopsis* Adaptation or Development-Dependent Cold Response

Cold stress-responsive proteins were predominantly accession-specific (Figure 6), and the observed differences supported the contrasting results of the stress response evaluation *via* determination of photosynthetic parameters and conductivity (Figures 4B–E). It is tempting to speculate that the results reflect adaptation of these accessions and documented high-impact mutations in the genes of the light signaling pathway (Supplementary Material S8). However, it should be noted that all accessions were cultivated for 4 weeks under conditions that were optimal for Col-0 growth, and differences in plants' phenotypes were visible (Figure 4A). A similar experimental setup has been previously employed in the evaluation of *Arabidopsis* accessions response to cold stress (e.g., Rasmussen et al., 2013; Cvetkovic et al., 2017; Leuendorf et al., 2020), and it cannot be excluded that at least part of the accession-specific response could be development-dependent.

### B20 Plants Accumulated More Cold-Stress Responsive Proteins

The comparison of B20 and R20 plants revealed 35 proteins that were more abundant in B20 plants, and the B20:R20 protein abundance ratio showed a positive correlation with the stress response documented by ion leakage and selected photosynthetic parameters (Figures 7B,D). It does not seem that these proteins would be negative markers of stress tolerance, and it is likely that the increased abundance only reflects a higher cold stress response. These proteins include chloroplast RNA-binding protein CP31A (AT4G24770) that stabilizes transcripts under cold stress conditions and may confer cold stress tolerance (Kupsch et al., 2012). Cold-induced modulation of membrane lipid composition is likely reflected in increased abundances of enzymes associated with fatty acid metabolism, including sn-2 acyl-lipid omega-3 desaturase (biosynthesis of 16:3 and 18:3 fatty acids; AT3G11170), long-chain acyl-CoA synthetase 1 (AT2G47240), 12-oxophytodienoate reductase 1 (AT1G76680), and 3-hydroxyacyl-CoA dehydrogenase (AT3G15290). Similarly, sucrose accumulation is a well-documented cold stress response (e.g., Novák et al., 2021) and correlates with the observed accumulation of sucrose biosynthetic enzyme (sucrose-phosphatase 2; AT2G35840).

The list of proteins with a higher abundance in B20 plants indicated a putative stimulatory effect on protein trafficking, including the coatomer subunit (AT1G62020) that mediates protein transport from the endoplasmic reticulum, protein AT5G16880 ESCRT (endosomal sorting complex required for transport), a subunit of clathrin-associated adapter protein complex HAP13 (AT1G60780), dynamin-related protein 13 (AT3G60190), secretion-associated protein SAR2 (AT4G0208), and a protein with a reported role in actin and tubulin folding (AT3G11830). Interestingly, two proteins indicated an increase in biotic stress defense mechanisms, namely, glucosinolate biosynthetic enzyme (indole glucosinolate O-methyltransferase 5; AT1G76790) and RNA-binding protein BTR1 that has a role in plant defense against virus infection (Fujisaki and Ishikawa, 2008).

### R20 Plants Invest More Resources Into the Chloroplast Development and Maintenance of Photosynthetic Apparatus

Protein analyses indicated that R20 plants possess an enhanced production of isoprenoids (1-Deoxy-d-xylulose 5-phosphate reductoisomerase, AT5G62790) without significant effect on cytokinin signaling (Figure 5), phytohormone auxin (Nitrilase 1, AT3G44310), and an osmoprotectant proline (rate-limiting enzyme Δ^1^-pyrroline-5-carboxylate synthase A, AT2G39800). The functional analysis also showed that proteins with a higher abundance in R20 plants mediate chloroplast development and photosynthesis (Figure 7C). The R20 plants had a lower abundance of an inhibitor of plastid division (MIND1, AT5G24020; Kanamaru et al., 2000), and accumulated multiple proteins essential for chloroplast biogenesis, development, and integrity, including CLPP6 protease (AT1G11750; Sjögren et al., 2006) and its protein interactor CLPC1 (AT5G50920), proteases FTSH1 and FTSH2 (AT1G50250, AT2G30950; Chen et al., 2006), calcium-sensing receptor CaS (AT5G23060; Huang et al., 2012), and protein translocase subunit SECA1 (AT4G01800; Skalitzky et al., 2011). An increase in abundance was found also for a protein required for chlorophyll accumulation under normal growth conditions (GUN4, AT3G59400; Larkin et al., 2003), a chloroplastic elongation factor required for proper chloroplast rRNA processing and/or translation at low temperature (AT5G13650, Liu et al., 2010), ferrochelatase regulating heme biosynthesis (AT2G30390), and multiple subunits of photosynthetic apparatus (Figure 7C). Taken together, the comparison of B20 and R20 plants showed that R20 plants invest more resources into the maintenance of efficient photosynthesis, and that is well in line with the measured photosynthetic parameters (Figures 4C–E). It is likely that the higher photosynthetic activity stimulates carbohydrate metabolism and starch production (a higher abundance of glucose-1-phosphate adenylyltransferase, AT5G19220) and could be responsible for an increase in ROS metabolism enzymes (ascorbate peroxidases AT1G7749, AT4G08390; peroxidase AT4G37520).

### Prolonged Cold Treatment Inhibits Cytokinin Signaling in a Light Quality-Independent Manner

Plant hormone cytokinin is well-known for its multifaceted function in growth and development (e.g., Skalák et al., 2019). Similarly, its role in abiotic stress is also well-documented (Pavlů et al., 2018). Previously, it was shown that a brief period of cold treatment upregulated cytokinin responsive genes but did not change the cytokinin content (Jeon et al., 2010). However, prolonged cold stress has a negative impact on the active cytokinin pool (Novák et al., 2021; Prerostová et al., 2021b). That is like other abiotic stressors (Zwack and Rashotte, 2015) and may reflect the growth arrest. The exact role of cytokinin in cold response and cold tolerance is far from understood. For instance, cytokinin receptors were shown to be negative regulators of freezing tolerance, but an increase in cytokinin pool by an exogenous application or promoted biosynthesis has a positive effect (Jeon et al., 2010; Prerostová et al., 2021b). Similarly, previous studies have reported cytokinin responsive genes ARR-A to be both positive and negative regulators of freezing tolerance (Jeon et al., 2010; Shi et al., 2012). Our previous results showed that cytokinin signaling in cold-treated plants is modulated by ambient light and that higher light intensity has a negative impact on its output (Novák et al., 2021). Surprisingly, B20- and R20-treated plants showed a somewhat similar gene expression profile (Figure 5) to that of plants treated with higher white light intensity (Novák et al., 2021). This profile was observed in all tested accessions suggesting a light quality-independent general attenuation of cytokinin signaling. The inhibition of cytokinin signaling is likely a growth regulatory mechanism restricting excess growth under suboptimal environmental conditions, and that is consistent with cytokinin/abscisic acid antagonism (Huang et al., 2018).

### Do Different Parts of the Light Spectrum Activate Specific Cold Tolerance Mechanisms?

The presented data demonstrate that *Arabidopsis* response to low temperature is strongly light-quality specific. The blue light treatment results in low temperature in higher expression of key cold-responsive transcription factors CBFs, higher accumulation of cold-stress responsive proteins, but more attenuated photosynthetic processes and increased oxidation status indicating a promoted ROS signaling. In contrast, red light inhibits CBF expression, but promotes the expression of chloroplast and ROS scavenging proteins, maintains photosynthetic processes, and decreases level of oxidation and membrane damage. Altogether, it seems that red light activates the program for maintaining cell function, and blue light induces a specific cold defense program. Neither of these mechanisms on their own can successfully protect plants from subsequent freezing damage, as seen by freezing stress tolerance. The W20 plants showed that the intensity of light is not the key limiting factor in this process, indicating the importance of complementarity in maintaining growth and specific cold protection mechanisms. Experiments with *Arabidopsis* accessions showed that the blue light perception *via* CRY2 is an integral part of cold response under blue light and its absence in Ws-0 (Supplementary Material S8) significantly altered plant physiology and proteome. Interestingly, it has been demonstrated that plants have dynamic life also in cold under the snowpack (reviewed in Körner, 2003). Robson and Aphalo (2019) found that the blue: red (B:R) ratio positively correlates with the depth of the snow surface. Taken together with the observations reported here, it seems that a low B:R ratio acts as a plant signal for promoting active photosynthesis and restoration of the vital processes (i.e., plant growing close to snowpack surface or in the melting snow), and an increase in B:R ratio signals less suitable conditions that require a stronger cold-specific response.

## Data Availability Statement

The datasets presented in this study can be found in online repositories. The names of the repository/repositories and accession number(s) can be found below: http://proteomecentral.proteomexchange.org/; PXD033102.

## Author Contributions

BB, RV, MC, and JN: conceptualization. MK: methodology and evaluation of the data, freezing assay, RT-qPCR, and physiology experiments. VO: physiology experiments. MC: proteomic analysis and evaluation of the data. LH: MDA analysis. MB: proteomic analysis. JN: physiology experiments and evaluation of the data. BB: evaluation of the data. MC, JN, MK, and BB: manuscript preparation. BB and RV: funding acquisition. All authors have read and agreed to the published version of the manuscript.

## Funding

Funding for this work was provided by (i) the Czech Science Foundation (Grants 17-04607S and 20-26232S), (ii) the Ministry of Education, Youth and Sports of the Czech Republic with support from the European Regional Development Fund (Grant No. CZ.02.1.01/0.0/0.0/16_019/0000738, project name Centre for Experimental Plant Biology), and (iii) the internal grant of Mendel University in Brno (AF-IGA-IP2018/030).

## Conflict of Interest

The authors declare that the research was conducted in the absence of any commercial or financial relationships that could be construed as a potential conflict of interest.

## Publisher's Note

All claims expressed in this article are solely those of the authors and do not necessarily represent those of their affiliated organizations, or those of the publisher, the editors and the reviewers. Any product that may be evaluated in this article, or claim that may be made by its manufacturer, is not guaranteed or endorsed by the publisher.
