# Supplementary figures and images for: Light Quality Modulates Plant Cold Response and Freezing Tolerance
Source: Front Plant Sci. 2022 Jun 9;13:887103. doi: 10.3389/fpls.2022.887103 (PMC9221075; doi:10.3389/fpls.2022.887103)

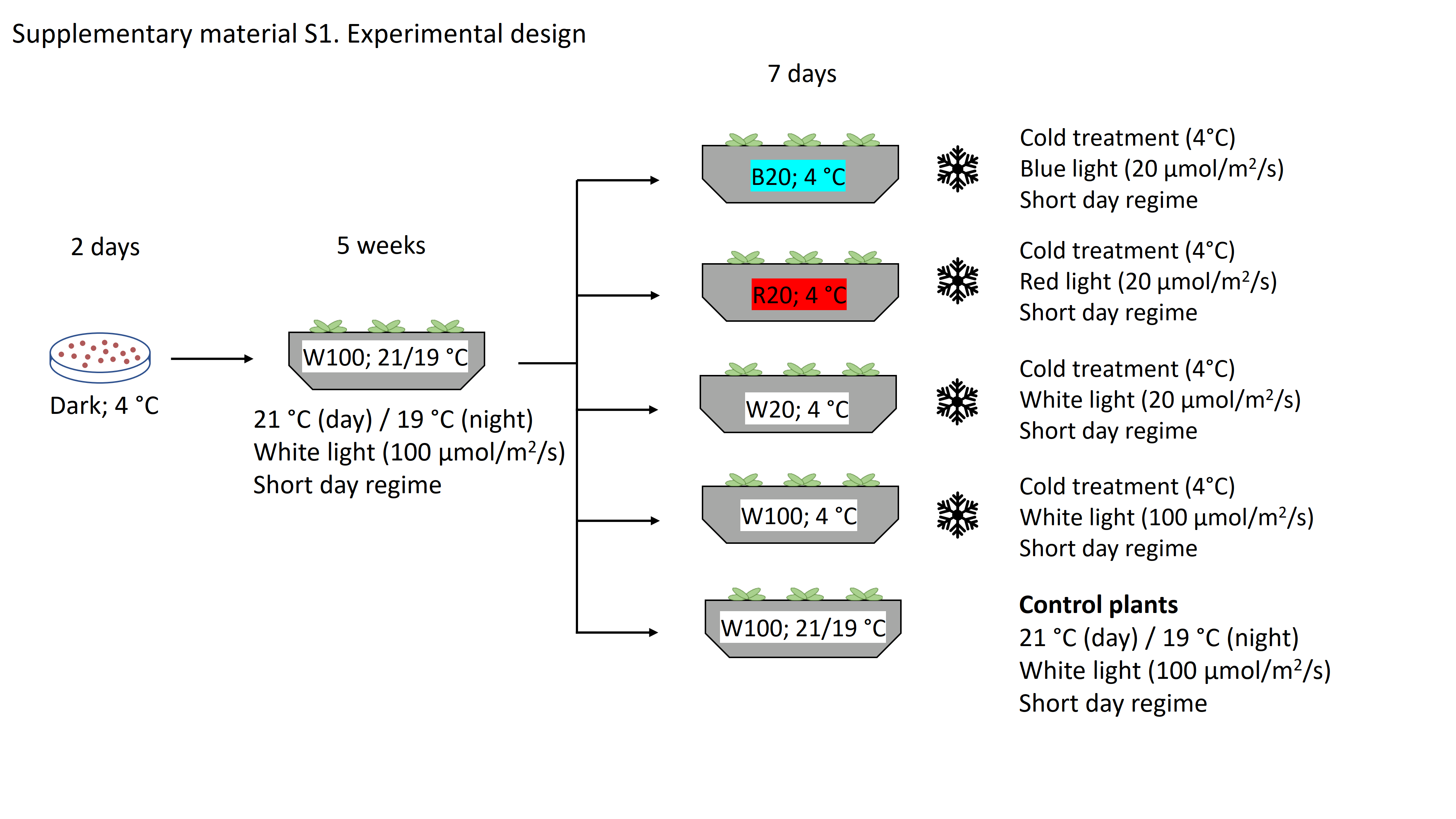

Supplement: Supplementary file 1 [file Image_1.tif]

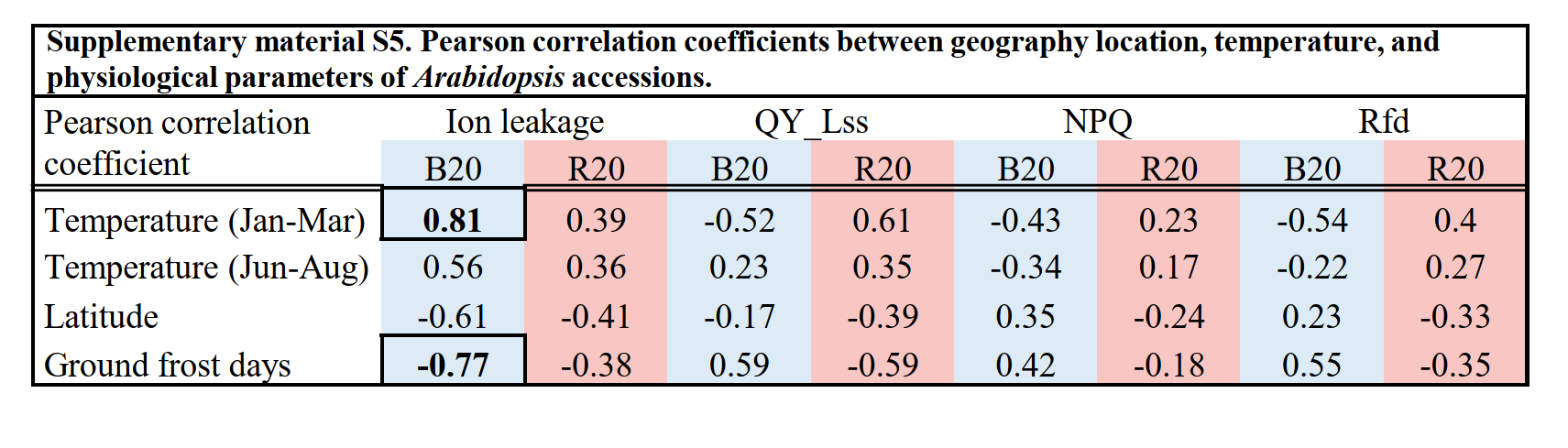

Supplement: Supplementary file 5 [file Image_2.TIF]
