# Supplementary material for: Light Quality Modulates Plant Cold Response and Freezing Tolerance
Source: Front Plant Sci. 2022 Jun 9;13:887103. doi: 10.3389/fpls.2022.887103 (PMC9221075; doi:10.3389/fpls.2022.887103)
Supplement: Supplementary file 2 [file Table_1.DOCX]

**Supplementary material S2. RNA and protein extraction protocol**

**Extraction buffer:** methanol:chloroform:water (2.5:1:0.5), [-20 °C]

**Wash buffer:** 0.75 % mercaptoethanol in methanol [-20 °C]

**Separation buffer:** chloroform:water (1:1)

**Solubilizing buffer:** 7 M guanidin:HCl; 2 % Tween-20; 4 % Triton-×100; 50 mM Tris; 1 % mercaptoethanol; pH 7.5 SDS buffer: 2 % SDS; 30 % sucrose; 5 % mercaptoethanol; 5 mM EDTA; 100 mM Tris; pH 8.0

**SDS buffer:** 2% (w/v) SDS, 30% (w/v) sacharosa, 5% (v/v) mercaptoethanol, 5 mM EDTA, 100 mM Tris, pH 8,0, [4°C]

**Precipitation solution:** 100 mM ammonium acetate in methanol [-20°C]

**RNA 1 wash buffer:** 2 mM Tris, 20 mM NaCl, 0,1 mM EDTA, 90% EtOH, pH 7,5

**RNA 2 wash buffer:** 2 mM Tris, 20 mM NaCl, 70% EtOH, pH 7,5

800 μl of extraction buffer was added to 20-50 mg of homogenized plant material, it was mixed and centrifuged for 6 minutes (4 ° C, 14,000 rpm). Supernatant was discarded. The pellet containing nucleic acids and proteins was covered with 1 ml of wash buffer, mixed and centrifuged for 6 minutes (4 ° C, 14,000 rpm). The supernatant was discarded and the washing step was repeated once more. After removing the supernatant, the remaining washing solution was allowed to evaporate. 400 μl of solubilizing buffer was added to the washed pellet, and the mixture was incubated for 20 minutes at 37 ° C and 800 rpm until the pellet was completely dissolved. This was followed by centrifugation for 4 minutes (4 ° C, 14,000 rpm). The supernatant was transferred to a silica gel column and centrifuged for 2 minutes (4 ° C, 8000 rpm). 450 μl of 100% acetonitrile (ACN) was added to the filtrate, then the solution was mixed and transferred to a new silica gel column, which was placed in a new microtube. Centrifugation was performed again for 2 minutes (4 ° C, 8,000 rpm), and the proteins were isolated from the infiltrate as described in the "Protein extraction" section (see below). RNA-binding columns were washed with 350 μl of RNA 1 wash buffer, centrifuged for 1 minute (4 ° C, 8,000 rpm). An 80 μl solution of 70 μl RDD buffer and 10 μl DNase (Qiagen, 1500 Kunitz units) was then applied to the column to remove any residual DNA. The column was incubated for 15 minutes at room temperature and then washed again with 400 μl of RNA 1 wash buffer. The sample was centrifugated for 1 minute (4 ° C, 8,000 rpm), the filtrate was removed and 750 μl of RNA 2 wash buffer was applied to the column. This was followed by centrifugation for 1 minute (4 ° C, 8,000 rpm), the filtrate was removed, and the column membrane was dried by centrifugation for 2 minutes (4 ° C, 10,000 rpm). RNA was eluted with 30 μl of RNase-free deionized water. The concentration of RNA obtained was measured spectrophotometrically using NanoDrop (Thermo Scientific, USA).

Protein extraction and preparation for LC-MS analyses

550 μl of phenol and 600 μl of LC-MS water was added to the protein-containing filtrate. It was mixed with a Retch Mill homogenizer for 45 seconds and centrifuged for 8 minutes (4 ° C, 10,000 rpm). Then, the resulting phenolic phase was transferred to a new microtube containing 600 μl of SDS buffer. It was again mixed with a Retch Mill homogenizer for 45 seconds and centrifuged for 8 minutes (20 ° C, 10,000 rpm). The resulting phenolic phase was transferred to a new microtube and made up to 2 ml with precipitation solution. Proteins precipitated overnight at -20 ° C. The next day, samples were centrifuged (5 min; 4 °C; 20,000×g), washed with 1 ml of 80% acetone (v/v) and centrifuged (5 min; 4 °C; 20,000×g). The supernatant was discarded and the pellet was air-dried and then resubilized in the solution of 50 mM ammonium bicarbonate (AMBIC) and 8 M urea at 25 °C for 60 minutes (Thermomixer comfort, Eppendorf, 600 rpm). Protein concentration was determined using Bradford assay (Sigma-Aldrich). Samples were diluted in solution of 50 mM ammonium bicarbonate (v/v) in 2.5% acetonitrile (ACN) and ddH2O (proportion 2:1) and digested overnight at 29 °C with with 1 µg of trypsin (Gold, Promega).Samples were acidified with 2% trifluoroacetic acid (TFA) and then desalted using C18 SPE columns (Agilent). The resulting tryptic peptides were concentrated on SpeedVac (Thermo).
